# Local and distal effects of arbuscular mycorrhizal colonization on direct pathway Pi uptake and root growth in *Medicago truncatula*

**DOI:** 10.1093/jxb/erv202

**Published:** 2015-05-04

**Authors:** Stephanie J. Watts-Williams, Iver Jakobsen, Timothy R. Cavagnaro, Mette Grønlund

**Affiliations:** ^1^School of Biological Sciences, Monash University, Clayton, VIC 3800, Australia.; ^2^Department of Chemical and Biochemical Engineering, Technical University of Denmark, 2800 Kgs. Lyngby, Denmark.; ^3^School of Agriculture, Food and Wine, The University of Adelaide, Waite Campus, PMB1, Glen Osmond, SA 5064, Australia.

**Keywords:** Arbuscular mycorrhizal (AM) fungi, colonized root patch, direct pathway uptake (DPU), *Medicago truncatula*, *mtpt4*-mutant, Pi transporters, resource allocation, soil P level.

## Abstract

Two pathways exist for plant Pi uptake from soil: via root epidermal cells (direct pathway) or via associations with arbuscular mycorrhizal (AM) fungi, and the two pathways interact in a complex manner. This study investigated distal and local effects of AM colonization on direct root Pi uptake and root growth, at different soil P levels. *Medicago truncatula* was grown at three soil P levels in split-pots with or without AM fungal inoculation and where one root half grew into soil labelled with ^33^P. Plant genotypes included the A17 wild type and the *mtpt4* mutant. The *mtpt4* mutant, colonized by AM fungi, but with no functional mycorrhizal pathway for Pi uptake, was included to better understand effects of AM colonization *per se*. Colonization by AM fungi decreased expression of direct Pi transporter genes locally, but not distally in the wild type. In *mtpt4* mutant plants, direct Pi transporter genes and the Pi starvation-induced gene *Mt4* were more highly expressed than in wild-type roots. In wild-type plants, less Pi was taken up via the direct pathway by non-colonized roots when the other root half was colonized by AM fungi, compared with non-mycorrhizal plants. Colonization by AM fungi strongly influenced root growth locally and distally, and direct root Pi uptake activity locally, but had only a weak influence on distal direct pathway activity. The responses to AM colonization in the *mtpt4* mutant suggested that in the wild type, the increased P concentration of colonized roots was a major factor driving the effects of AM colonization on direct root Pi uptake.

## Highlight

Mycorrhiza effects on the direct pathway for plant phosphorus uptake were modulated by mycorrhizal phosphorus input to the roots, rather than by mycorrhizal colonization itself.

## Introduction

Phosphate rock as a fertilizer is important for crop production worldwide. It is a limited and non-renewable natural resource, being depleted at an increasing rate, while demand for food production also increases (Cordell *et al.*, 2009; Gilbert, 2009). Soil inorganic phosphate (Pi) is essential for the proper growth and functioning of plants, and plant growth and yield are adversely affected when Pi supply is limited (Epstein and Bloom, 2005; Marschner and Marschner, 2012). Therefore, maximizing the efficiency of plant acquisition of soil P with limited or no fertilizer application is important for the future of agriculture (López-Arredondo *et al.*, 2014).

Root epidermal cells and roots hairs take up Pi from the soil via the direct pathway for Pi uptake (DPU), and this creates a nutrient depletion zone around the root due to the low mobility of P in soil (Smith *et al.*, 2011). Furthermore, plants need to overcome a large electrochemical potential gradient in order to move Pi into cells (Schachtman *et al.*, 1998). As a consequence, plants have evolved strategies that can increase the acquisition of root-derived P (Lambers *et al.*, 2010). One strategy for foraging for nutrients beyond the depletion zone of roots is the formation of arbuscular mycorrhizas, an association between the plant and specialized soil fungi (Smith and Read, 2008). Arbuscular mycorrhizas increase plant uptake of soil nutrients such as P, Zn, Ca, and Cu when they are in limited supply, thus improving plant nutrition (Marschner and Dell, 1994). The formation of mycorrhizas creates an arbuscular mycorrhizal (AM) pathway for Pi uptake (MPU), which interacts with the DPU (Smith *et al.*, 2003, 2004). Pi uptake via the MPU is often reduced with increasing soil P concentration, but this effect is influenced by the AM fungal species (Facelli *et al.*, 2014).

While the formation of mycorrhizas can greatly enhance plant P acquisition, and thence plant growth, there are some instances where mycorrhizas do not appear to provide a benefit in terms of growth or P nutrition (Grace *et al.*, 2009). The AM pathway can still be active, but ‘hidden’, when an AM growth or P response is absent, and can actually dominate Pi uptake (Smith *et al.*, 2003; Nagy *et al.*, 2009). Previously, mycorrhizas have been shown to contribute up to 100% of a plant’s P (Smith *et al.*, 2004). Thus, the activity of the AM pathway when no growth or P response is observed cannot be discounted (Poulsen *et al.*, 2005; Bucher, 2007), and further investigation into this area is needed, especially into the interplay between the DPU and MPU, at both molecular and physiological levels (Smith *et al.*, 2011).

Root Pi transporter genes involved in the direct P uptake pathway can be down-regulated in AM plants, independent of the external P status or extent of AM colonization (Paszkowski *et al.*, 2002; Glassop *et al.*, 2005; Harrison, 2005; Christophersen *et al.*, 2009). The genes that encode both the direct and AM Pi transporters have been described in many plant species, including *Medicago truncatula* (H. Liu *et al.*, 1998; Javot *et al.*, 2007; Liu *et al.*, 2008). The direct pathway Pi transporter genes *MtPT1, MtPT2*, and *MtPT3* are closely related low-affinity Pi transporters, belonging to the PHT1 family (Bucher *et al.*, 2001; Liu *et al.*, 2008). The phosphate transporter gene *MtPT1* is an important direct Pi transporter gene that appears to be expressed exclusively in the root epidermal cells, cortex, and root hairs (Chiou *et al.*, 2001; Liu *et al.*, 2008). Transcript and protein levels are closely correlated for *MtPT1*, and increase greatly in response to Pi starvation (Chiou *et al.*, 2001). Furthermore, *MtPT1* expression levels (transcripts and protein) decrease when the plant becomes colonized by AM fungi (Chiou *et al.*, 2001; Liu *et al.*, 2008). The phosphate transporter gene *MtPT2* acts in a similar way to *MtPT1* with regards to Pi fertilization and AM colonization (H. Liu *et al.*, 1998). However, promoter activity for *MtPT2* is strongly located in the vascular cylinder (Xiao *et al.*, 2006), indicating a role in plant P translocation. The direct phosphate transporter gene *MtPT3* is down-regulated by Pi fertilization, and in roots colonized by three species of AM fungi (Grunwald *et al.*, 2009), and is suggested to be expressed in the vascular tissue of roots (Liu *et al.*, 2008). Two other direct Pi transporter genes have been identified, also belonging to the PHT1 family: *MtPT5*, a high-affinity Pi transporter, expressed in all parts of the root except the vascular tissue, and affected only by some AM fungi; and *MtPT6*, which is unaffected by Pi, but has reduced expression in AM plants (Grunwald *et al.*, 2009). Thus, numerous Pi transporter genes are working to transport soil Pi directly via the roots in *M. truncatula*.

Of particular importance to the present study is the low-affinity Pi transporter MtPT4. This protein has been identified as a mycorrhiza-induced Pi transporter in *M. truncatula* (Harrison *et al.*, 2002; Javot *et al.*, 2007). The gene encoding MtPT4 is exclusively expressed in AM roots, specifically in cells containing arbuscules (Harrison *et al.*, 2002; Grunwald *et al.*, 2009). Loss of the MtPT4 function is detrimental to the AM symbiosis because it causes mature arbuscules to degenerate, leading to premature arbuscule death (Javot *et al.*, 2007). Arbuscule development can be restored in the *mtpt4* mutant, under low soil N conditions (Javot *et al.*, 2011).

Expression of direct Pi transporter genes in non-mycorrhizal plants is regulated via Pi starvation signalling pathways, which also regulate various other signalling components. While most of the signalling components of Pi starvation responses have been described in the non-mycorrhizal model plant species *Arabidopsis thaliana*, many are also present in AM plants (Smith *et al.*, 2011). Phosphate starvation-induced (PSI) genes are important components of the Pi starvation regulatory pathways, along with Pi transporters, phytohormones, transcription factors, and microRNAs (Rouached *et al.*, 2010). A PSI gene in *M. truncatula*, *Mt4* (Burleigh and Harrison, 1997), is down-regulated in roots by Pi fertilization and AM colonization (Burleigh and Harrison, 1999). Plant responses to Pi-depleted soil can occur locally via changes in root system architecture (e.g. root hair formation), as well as systemically (long-distance) through phosphate homeostasis (Martín *et al.*, 2000; Franco-Zorrilla *et al.*, 2004; Péret *et al.*, 2011). Additionally, plant responses to Pi starvation can be physiological or molecular in nature, such as changes to the allocation of resources (photosynthate) and regulation of PSI genes (Raghothama, 1999). The inhibition of AM colonization by high soil P levels works systemically, as it is linked to shoot P concentrations rather than local P concentrations in roots (Breuillin *et al.*, 2010; Balzergue *et al.*, 2011).

It has previously been suggested that mycorrhizas may have a limited long-distance impact upon the direct Pi pathway (Grønlund *et al.*, 2013). This suggestion was based on experiments with *Pisum sativum* (pea) plants that were grown at just one soil P level (7.5mg P kg^–1^ soil) and were incompletely investigated for expression of direct Pi transporter genes (*PsPT1* only). Therefore, it was not possible to conclude if the mycorrhiza-related effect on the direct Pi uptake pathway resulted from an increased P status in AM plants or from more direct interactions between the AM fungus and the DPU (Smith *et al.*, 2011).

Those limitations in Grønlund *et al.* (2013) were addressed in the present study with *M. truncatula* in split-root systems. In addition to the A17 wild type, the *mtpt4* mutant *M. truncatula* genotype (Harrison *et al.*, 2002; Javot *et al.*, 2007) served to separate the effects of plant Pi signalling and of AM colonization on the regulation of plant P uptake by the direct and AM pathways. Furthermore, three soil P levels served to investigate that regulation in terms of shoot P status.

The hypotheses tested were (i) that localized root colonization by AM fungi will reduce DPU activity locally but not in a distal, non-colonized patch of root; (ii) that mycorrhiza-induced effects on DPU activity can be separated into P-derived and direct AM effects by comparing the wild-type and *mtpt4* genotypes; and (iii) that colonized and non-colonized patches of roots will experience differences in root growth and P nutrition.

## Materials and methods

### Soil and plants

**Fig. 1.**
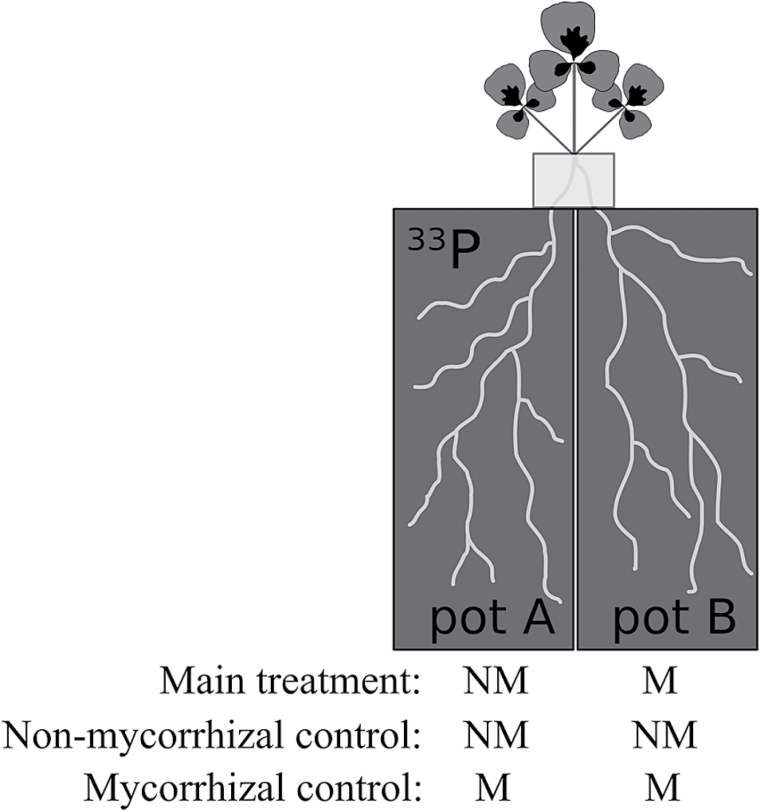
Set up of split-pots and AM fungal inoculation treatments (not to scale). The left-hand pot (pot A) had unlabelled soil layers at the bottom (150g) and top (100g) of the pot, and 600g of soil labelled with ^33^P in between the two unlabelled layers; see the Marerials and methods for further details. NM, non-mycorrhizal; M, mycorrhizal.

The *M. truncatula* cultivar Jemalong A17 (referred to as the wild type hereafter) and mutant *mtpt4-1* were used in this study, kindly provided by Maria Harrison. Seeds of both genotypes were scarified, surface-sterilized, imbibed, and pre-germinated (following Grunwald *et al.*, 2009). Pre-germinated seeds were sown (day 0) in small pots containing 160g of a semi-sterile (15 kGy, 10 MeV electron beam) 1:1 soil/sand mix, which was supplemented with basal nutrients (as per Merrild *et al.*, 2013) including 10mg P kg^–1^ (as KH_2_PO_4_), and mixed thoroughly. Seedlings grew for 16 d before being moved to split-pots (following Grønlund *et al.*, 2013). Shoot and root P contents were 0.04mg and 0.04mg P plant^–1^ in wild-type plants, respectively, and 0.06mg and 0.05mg P plant^–1^ in *mtpt4* plants, on the day of transfer. The root system of each plant was divided into two halves of a similar size and planted in a split-root system consisting of two adjoining pots with one root half in each (see Fig. 1). Pots contained a semi-sterile 1:1 soil/sand mixture, as above. Three soil P concentrations were established by mixing KH_2_PO_4_ into the growth medium at 0, 20, and 50mg P kg^–1^. These P additions resulted in levels of 0.5M NaHCO_3_-extractable P (Olsen *et al.*, 1954) of 5.0±0.2, 10.1±0.3, and 19.9±0.9mg P kg^–1^ soil and are referred to as P0, P20, and P50 hereafter. While the *M. truncatula* wild type was grown in all soil P treatments, the mutant *M. truncatula mtpt4* was grown at P20 only.

The split-root treatments were (i) M,M with AM fungal inoculum in both root halves; (ii) NM,M with one root half non-inoculated and the other inoculated with AM fungi; and (iii) NM,NM with both root halves non-inoculated (see Fig. 1). Soil in M pots was mixed with 75g of *Rhizophagus irregularis* (previously named *Glomus intraradices*, Schüßler and Walker, 2010) BEG87 inoculum, consisting of a mixture of dry soil, spores, and root fragments of a *Trifolium subterraneum* pot culture. Pot A contained 600g of soil mixed with 0.5 kBq g^–1^ of carrier-free H_3_^33^PO_4_. The specific activity in 0.5M NaHCO_3_ extracts of the ^33^P-labelled P0, P20, and P50 soils was 29.7±2.7, 12.4±1.5, and 7.1±0.5 kBq mg P^–1^, respectively. Isotope labelling was used to quantify the Pi uptake from pot A.

A layer of unlabelled, non-inoculated soil was weighed into both the bottom (150g) and top (100g) of each pot, to prevent transfer of labelled soil and AM fungal inoculum between pots. There were five replicates of each treatment. Plants were grown in climate chambers with a 16/8h light/dark cycle at 20/15 °C. Fluorescent daylight lamps (Osram GmbH, Munich, Germany) provided 500 μmol m^–2^ s^–1^ photosynthetically active radiation (PAR; 400–700nm). Pots were watered every second day to 60% of water-holding capacity.

### Harvesting and physiological analysis

Plants were harvested 37 d after sowing (21 d after transplantation to split-pots). A subsample (~50mg) of fresh shoot material was flash-frozen in liquid N_2_ and kept at –80 °C. Shoots were dried at 70 °C for 48h and dry weights recorded. Roots were washed, blotted, and weighed, and a weighed subsample for determination of AM colonization was stored in 50% ethanol. Another ~500mg subsample of root material was flash-frozen in liquid N_2_, crushed, and kept at –80 °C for RNA isolation. The remaining root tissue was dried at 70 °C for 48h and dry weights were determined.

Dried shoot and root samples were oxidized in a 4:1 mixture (v:v) of 65% nitric:70% perchloric acids, and total P was determined by the molybdate blue method using AutoAnalyzer 3 (Bran+Luebbe, Norderstedt, Germany). The ^33^P in shoot and root tissue was determined in the same digests in a Packard TR 1900 liquid scintillation counter (PerkinElmer, Waltham, MA, USA). Shoot Pi concentrations were analysed after homogenizing ~50mg of frozen sample in 1ml of 1M HCl and centrifugation for 3min at 14 000 *g*. A 15 μl sample of the supernatant was mixed with 240 μl of malachite green (0.045% w/v)/ammonium molybdate (4.2% w/v) solution in a microplate. After 1min, 30 μl of 34% (w/v) sodium citrate was added and the OD_660_ was read 5–10min later (modified from Lanzetta *et al.*, 1979). Total N in shoot samples was determined in 2mg of finely homogenized samples on an EA 1110 elemental analyser (Thermo Scientific, Bremen, Germany).

Prior to determining the percentage root length colonized by AM fungi, root samples were cleared in 10% KOH and stained with trypan blue following Kormanik and McGraw (1982). All roots were then assessed for total percentage root length colonized by AM fungi, and root length (Newman, 1966). Additionally, the proportion of the root containing AM structures (arbuscules, vesicles, and/or internal hyphae) was assessed on subsamples of stained roots from pot B of the NM,M pots at P20 only (McGonigle *et al.*, 1990). The presence of hyphae was investigated in root-free HC soil taken from pot B of the NM,M P20 treatments, for both the wild-type and *mtpt4* mutant plants, and also in HC soil from the equivalent NM,NM pots. Qualitative information was obtained by stereo microscope inspection of moist soil, while quantitative data were obtained by measuring the length of hyphae collected on membrane filters (Jakobsen *et al.*, 1992).

### RNA isolation and real-time qPCR analysis

Total RNA was extracted from ~100mg of root samples of *M. truncatula* wild type and of mutant *mtpt4* grown at 20mg P kg^–1^ soil, using an miRNeasy Mini Kit (Qiagen Hilden, Germany) with on-column DNase treatment following the manufacturer’s protocol. RNA concentration was measured on a Nanodrop ND-1000 Spectrophotometer (Saveen 1 Werner, Malmö, Sweden). cDNA was synthesized from 200ng of total RNA using dNTP Mix (Qiagen) and Expand Reverse Transcriptase (Roche) including Protector RNase inhibitor (Roche).

The real-time PCR primers (Eurofins MWG operon, Germany) were *EF1α* (Liu *et al.*, 2008), *MtPT1, MtPT2, MtPT3, MtPT4* (Grunwald *et al.*, 2009), and *Mt4* (Branscheid *et al.*, 2010). Gene expression analysis was carried out on four replicate plants from each treatment. Real-time PCR analysis was performed using the Rotor Gene 2000 Real Time Cycler (Qiagen). Each 20 μl of PCR contained 8 μl of a 1.25^–1^ dilution of the reverse transcription reaction (see above), and 12 μl of SYBR Green Master Mix (Fermentas, Thermo Scientific), which included 500nM of each primer. Samples were heated to 95 **°**C for 10min, followed by 40 cycles of 15 s at 95 **°**C and 1min at 60 **°**C. After each PCR, the specificity of the amplification was verified by running a melt curve analysis. The Rotor Gene 2000 software calculated relative amounts of RNA based on PCR cycle threshold values obtained from a dilution series from 2.5^–1^ to 6.25^–3^ (each step was a 1:3 dilution in H_2_O) of a standard reverse transcription sample from a mycorrhizal or non-mycorrhizal plant (depending on the primer of interest). Data were normalized to *EF1α* mRNA levels.

### Calculations

In the NM,NM and M,M control treatments, the shoot P derived from pot A at each soil P level could be directly derived as 50% of the measured shoot P content:

$$\begin{array}{l} \text{Measured P uptake from pot A}=\\ 0.\text{5}\times \left(\begin{array}{l} \text{shoot P content at harvest}\\ –\text{shoot P content in seedlings at planting} \end{array}\right) \end{array}$$ (1)

Shoot P derived from pot A could also be estimated from the ^33^P data:

$$\begin{array}{l} \text{Estimated P uptake from pot A}=\\ \left({}^{\text{33}}{\text{P}}_{\text{shoot}}/{\text{SA}}_{\text{33P in NaHCO3 extracts}}\right)\times \text{85}0/\text{6}00 \end{array}$$ (2)

The numerical ratio corrects for the fact that ^33^P was mixed with only 600g of the 850g soil in total. While the measured and the estimated P uptake from pot A increased with increasing P level as expected, the two methods produced different results within each P level. Thus the ratios between estimated and measured P uptake were 0.41, 0.77, and 0.88 at P0, P20, and P50, respectively. These ratios are means of very similar values in the NM,NM and M,M treatments at a given soil P level and it was therefore assumed that the observed deviation was not caused by the mycorrhizal status of the roots, but rather by P level-associated differences in isotopic equilibrium between ^33^P and ^31^P in the ‘plant available’ soil P pool. Due to the similar ratios for M,M and NM,NM plants, the ratios could be used to calculate the shoot P derived from pot A (i.e. the NM root half) in the NM,M treatments, and at a given P level by dividing the estimated uptake (Equation 2) by one of the three ratios given above.

Shoot uptake of P from pot B was derived as the difference between total shoot P and shoot P derived from pot A, and then the root length-specific (RL spec) P uptake from pot B was calculated. The minimum contribution by the MPU to the root length-specific uptake from pot B in the M,M and NM,M treatments was estimated by subtracting the corresponding NM,NM treatment values. The minimum percentage contribution of mycorrhizas to root length-specific P uptake from pot B was then calculated:

$$\begin{array}{l} \text{Minimum }\%\text{ AM contribution to shoot P}=\\ \text{1}00\times (\text{RL spec P uptake}\left(M\right)–\text{RL spec P uptake}\\ \left(\text{NM,NM}\right)/\text{RL spec P uptake}\left(M\right) \end{array}$$ (3)

The calculation for P contribution via the MPU in NM,M plants was modified from Smith *et al.* (2004), due to the disparity between estimated and measured MPU uptake in the mycorrhizal and non-mycorrhizal control plants when using the original equation.

### Statistical analysis

Two-way analyses of variance (ANOVA) were performed on the data, with pot configuration and soil P level as factors when analysing the wild-type genotype only, and pot configuration and genotype as factors when comparing the wild-type and *mtpt4* mutant genotypes at P20 only (see Supplementary Table S1 available at *JXB* online for ANOVA outcomes). One-way ANOVA were performed on wild-type distribution data (ratios) only. Where significant (*P*<0.05) interactions or main effects were found, comparisons were made using Tukey’s honestly significant difference (HSD). Student’s *t*-tests were performed for certain response variables (specified in the Results). All statistical analyses were performed using JMP 11.2.0 (SAS Institute Inc.).

## Results

### Root colonization and extraradical hyphal length

**Fig. 2.**
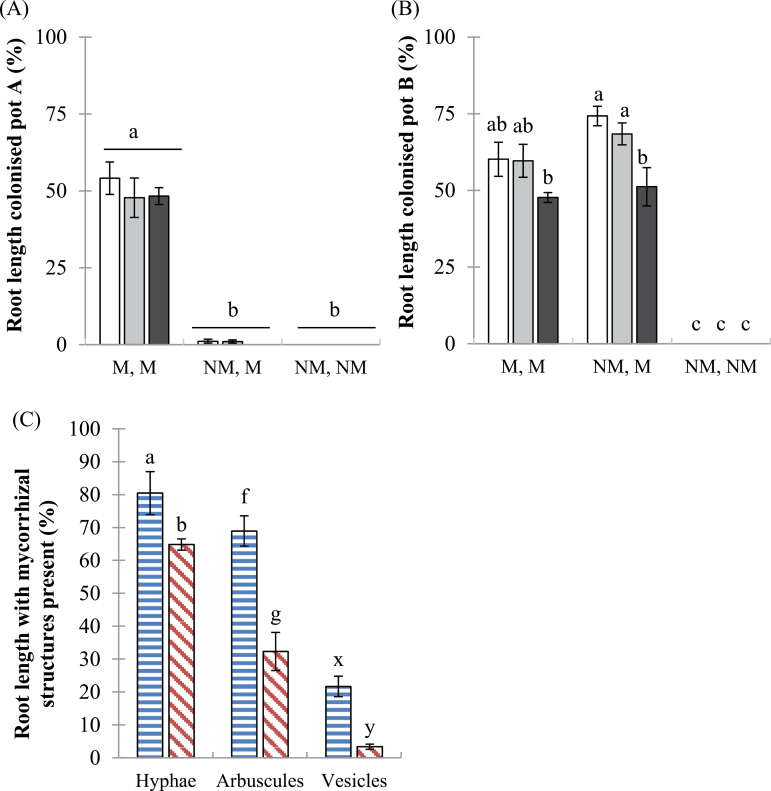
Mycorrhizal colonization in wild-type and *mtpt4* roots. Root length colonized (%) in the wild-type genotype in (A) pot A and (B) pot B, at three soil P additions: 0 (white bars), 20 (grey bars), and 50 (black bars) mg P kg^–1^. Values are the mean ±standard error, *n*=5. Means followed by the same letter were not significantly different at the *P*<0.05 level (Tukey’s HSD). (C) Presence of different mycorrhizal structures (hyphae, arbuscules, and vesicles) within roots grown at P20 in pot B of NM,M pots only. Wild type, horizontal bars; *mtpt4* mutant, diagonal bars. For (C) a different set of letters (ab, fg, xy) indicated a significant difference (at the *P*<0.05 level, Student’s *t*-test) between genotypes, for each type of colonization separately. (This figure is available in colour at *JXB* online.)

Root length colonized by AM fungi was <1% in all NM pots whereas roots in M pots had 48–74% of their root length colonized (Fig. 2A, B). Mycorrhizal colonization decreased significantly with increasing soil P in the pot B roots of NM,M pots (Supplementary Table S1A at *JXB* online). Mycorrhizal colonization in the *mtpt4* mutant was 32–36% of the root length and was thus lower than in the wild-type genotype (Supplementary Table S2). Furthermore, hyphal, arbuscular, and vesicular colonization in pot B of the NM,M pots were each significantly higher in the wild-type genotype than in the *mtpt4* mutant, with the greatest effects on arbuscule and vesicle content (Fig. 2C).

External hyphae of AM fungi were found in soil from both colonized wild-type and *mtpt4* mutant treatments, although the abundance was significantly lower in the latter (7.03±0.72 and 3.63±0.41 m g^–1^ dry soil, respectively). Although quantitative analysis did not reveal a significant difference in hyphal length density between *mtpt4* mutant soil and NM soil (2.79±0.50 m g^–1^ dry soil), direct microscopy confirmed the presence of hyphae in moist HC soil from *mtpt4* mutant plants, but not from non-mycorrhizal plants.

### Plant growth responses to soil P level and mycorrhizal colonization of one or both root halves

**Fig. 3.**
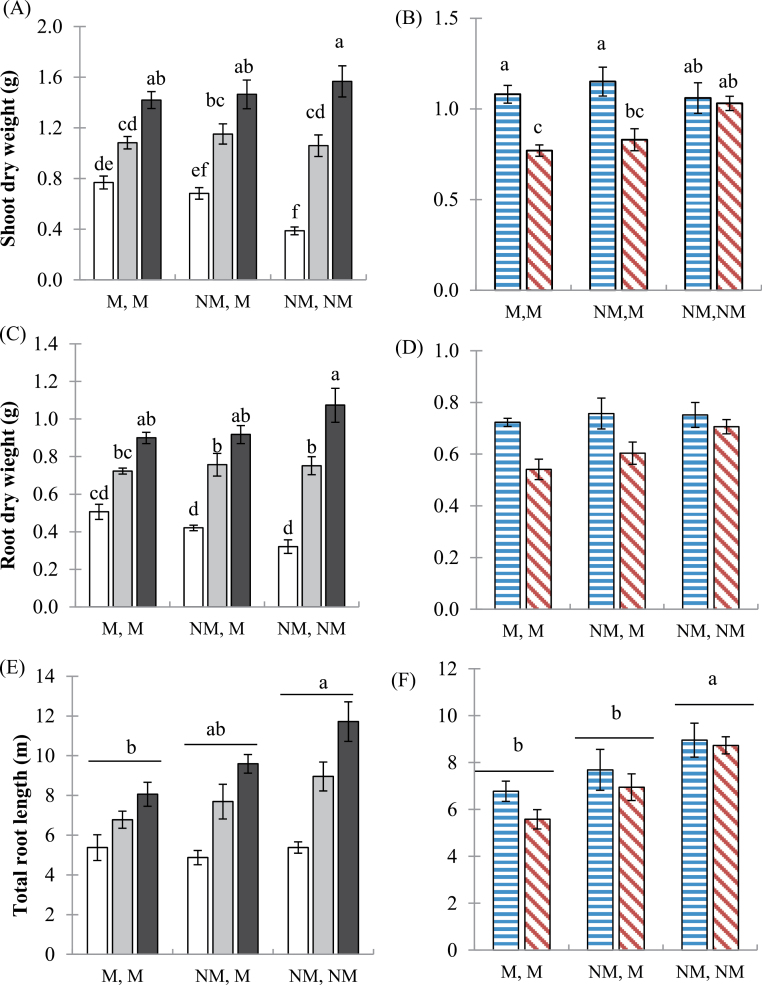
Effects of soil P level and AM colonization on plant growth. Shoot and root dry weights and total root length (pot A and pot B) of (A, C, E) the wild-type genotype at three soil P additions: 0 (white bars), 20 (grey bars), and 50 (black bars) mg P kg^–1^, and (B, D, F) the wild type (horizontal bars) and *mtpt4* mutant (diagonal bars) at P20 only. Values are the mean ±standard error, *n*=5. Means followed by the same letter were not significantly different at the *P*<0.05 level. (This figure is available in colour at *JXB* online.)

The wild-type plants exhibited strong positive growth responses to formation of mycorrhizal associations and to soil P supply. Shoot and root growth, and total root length of the wild-type genotype increased significantly over three levels of P addition: 0, 20, and 50mg P kg^–1^ (Fig. 3A, C, E). Furthermore, at low P (P0), shoot growth was significantly higher in the mycorrhizal pots (M,M) than the non-mycorrhizal pots (NM,NM). There was no such pattern at the higher soil P additions. The *mtpt4* plants had significantly lower root dry weights than the wild-type plants (pooling pot configuration), and significantly lower shoot dry weights when one or both pots were mycorrhizal (Fig. 3B, D). Total root length in the wild-type plants was significantly lower in the mycorrhizal pots than in the non-mycorrhizal pots, pooling soil P addition treatments (Fig. 3E), but this effect disappeared at 0mg P kg^–1^ (Supplementary Table S1 at *JXB* online: significant interaction between P level and pot configuration). Colonization by AM fungi also resulted in reduced root length in *mtpt4* mutant plants (Fig. 3F). Importantly, when both pot halves were non-colonized, the wild-type and *mtpt4* plants exhibited similar physiological responses, and hence there were no pleiotropic effects of the *mtpt4* mutation.

The distribution of resources between colonized and non-colonized root-halves (i.e. NM,M pots) was calculated as ratios of root biomass or root length in the colonized pot (M) to the total biomass or length (NM+M). Importantly, in the NM,NM and M,M pots, the contribution to root biomass and length was equal between both root halves as expected, in both wild-type and *mtpt4* plants; that is, the ratio was ~0.5 (data not shown).

**Fig. 4.**
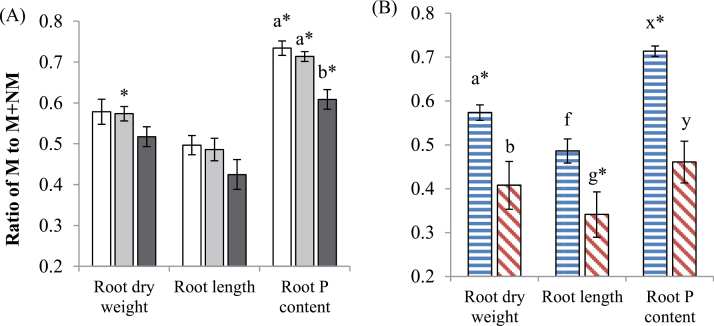
The distribution of resources between colonized and non-colonized root halves (i.e. NM,M pots) was calculated as the ratios of the colonized pot (M) to the total (NM+M). Ratios for root dry weight, length, and P content in (A) wild-type plants at three soil P additions: 0 (white bars), 20 (grey bars), and 50 (black bars) mg P kg^–1^, and in (B) the wild type (horizontal bars) and *mtpt4* mutant (diagonal bars) at P20 only. Values are the mean ±standard error, *n*=5. Means followed by the same letter were not significantly different at the *P*<0.05 level (Tukey’s HSD). For (B) different sets of letters (ab, fg, xy) indicate a significant difference (at the *P*<0.05 level, Student’s *t*-test) between genotypes, for different response variables, respectively. Asterisk denotes values that are significantly different from 0.5. (This figure is available in colour at *JXB* online.)

In the NM,M-grown wild-type plants, the ratio for both root length and biomass tended to decrease with increasing soil P concentration, but not significantly (*P*>0.05, one-way ANOVA, Tukey’s HSD, Fig. 4A). This indicated that the AM root half contributed less to root biomass accumulation as the soil P addition increased. At P20, the calculated ratio for root biomass and root length was significantly higher in the wild-type than in the *mtpt4* mutant genotype (*P*<0.05, Student’s *t*-test, Fig. 4B). In particular, the ratio was <0.5 for both response variables in the *mtpt4* genotype, indicating that the AM root half contributed less than half of the total root biomass and length.

### Responses in plant P nutrition to soil P level and mycorrhizal colonization of one or both root halves

**Fig. 5.**
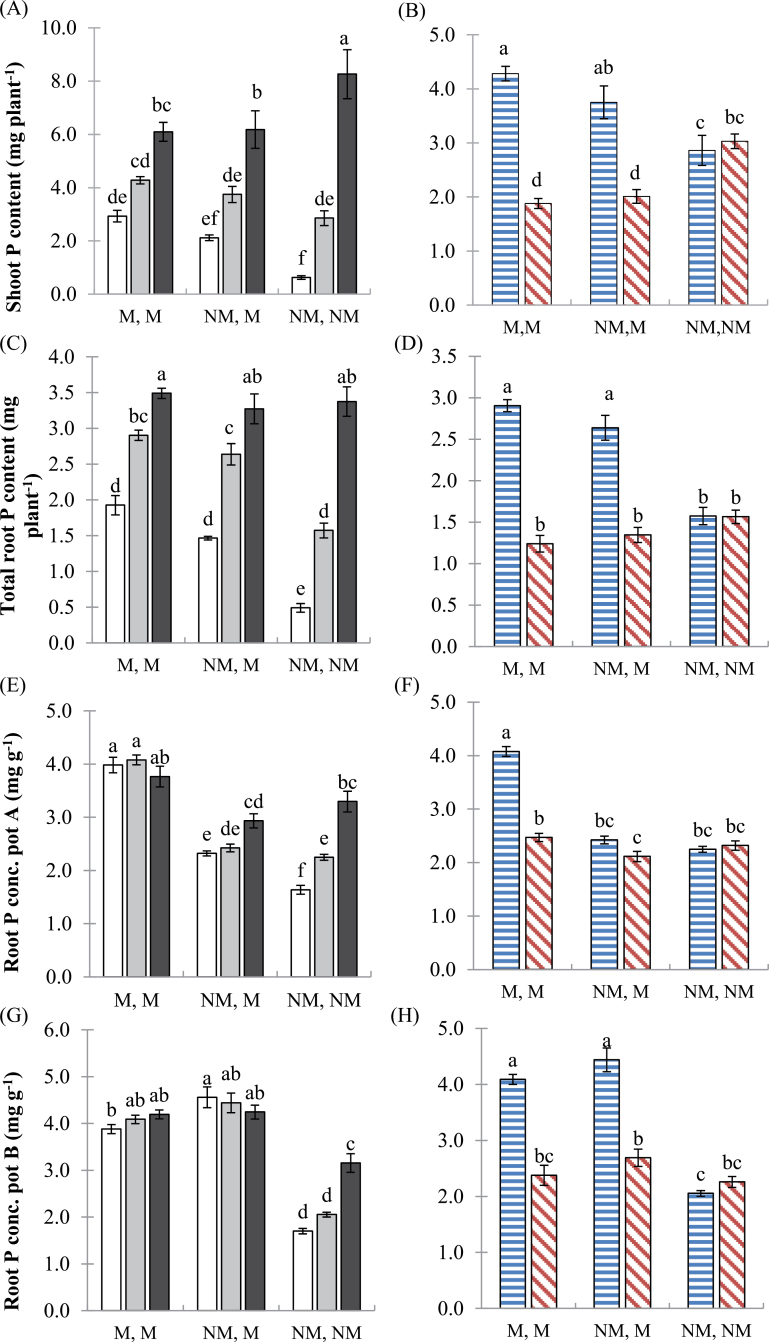
Effects of increasing soil P and AM colonization on plant P content and concentration. Shoot and root P content (mg plant^–1^), and root P concentration in pot A and pot B (mg g^–1^) of (A, C, E, G) the wild-type genotype at three soil P additions: 0 (white bars), 20 (grey bars), and 50 (black bars) mg P kg^–1^, and (B, D, F, H) the wild type (horizontal bars) and *mtpt4* mutant (diagonal bars) at P20 only. Values are the mean ±standard error, *n*=5. Means followed by the same letter were not significantly different at the *P*<0.05 level. (This figure is available in colour at *JXB* online.)

As for biomass, shoot and root P content as well as root P concentration in the wild-type plants responded strongly to AM colonization and soil P. Shoot and root P content increased significantly with increasing soil P addition and, at low P (P0), were significantly higher in the mycorrhizal than in the non-mycorrhizal wild-type plants (Fig. 5A, C). In the *mtpt4* genotype at P20, shoot and root P contents were similar to those of the wild type for completely non-mycorrhizal plants, and were otherwise significantly lower than in the wild type (Fig. 5B, D).

Ratios describing the distribution of P content between the non-mycorrhizal and mycorrhizal root halves of NM,M plants were calculated as for biomass. In the wild-type plants, the allocation of P to the mycorrhizal pot was significantly higher than 0.5 at all soil P addition treatments; however, it decreased significantly as soil P addition increased (*P*<0.05, one-way ANOVA, Tukey’s HSD, Fig. 4A). In the *mtpt4* mutant plants, allocation of P content was even between the mycorrhizal and non-mycorrhizal root halves, and the wild-type plants allocated significantly more P to the mycorrhizal half than the *mtpt4* plants (*P*<0.05, Student’s *t*-test, Fig. 4B).

Root P concentrations in the colonized wild-type roots were not significantly different at varying soil P additions. Conversely, non-colonized wild-type roots responded positively and significantly to soil P addition (Fig. 5E, G). Concentrations of P in the roots (both pot A and B) of *mtpt4* plants were similar between pot configurations, and were significantly lower than those of the mycorrhizal wild-type roots (Fig. 5F, H).

Shoot PO_4_ concentration increased significantly with increasing soil P concentration in the wild-type plant (pooling pot configuration), and was significantly lower in the non-mycorrhizal (NM,NM) pots than in the mycorrhizal (M,M) pots (pooling soil P addition, Supplementary Fig. S1A at *JXB* online). At P20, wild-type plants grown in the M,M pots had significantly higher shoot PO_4_ concentration than wild-type and *mtpt4* plants of other treatments, except the non-mycorrhizal wild-type treatment (Supplementary Fig. S1B).

**Fig. 6.**
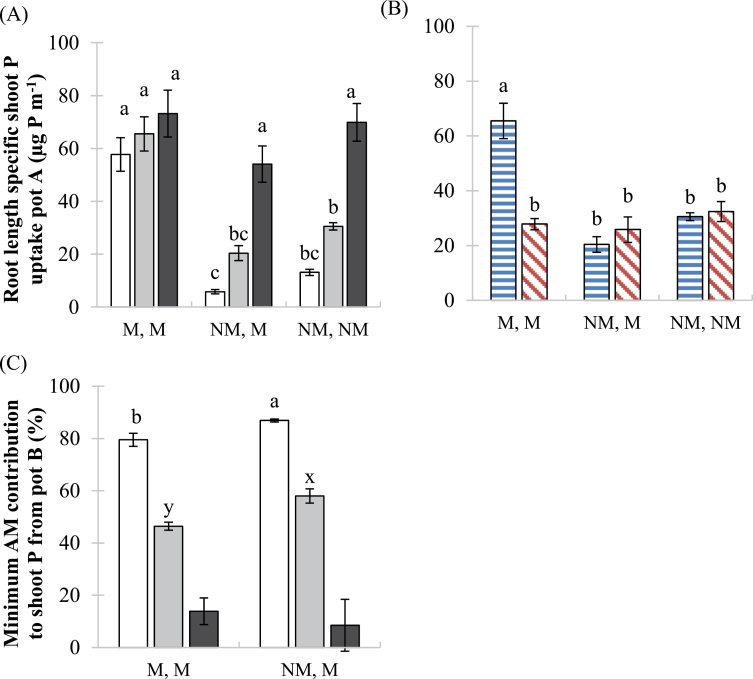
Pi uptake via the direct Pi uptake pathway and the mycorrhizal pathway. (A) Root length-specific shoot Pi uptake from pot A and (C) the minimum contribution by AM fungi from pot B to shoot P of wild-type plants at three soil P additions: 0 (white bars), 20 (grey bars), and 50 (black bars) mg P kg^–1^. (B) Root length-specific shoot Pi uptake from pot A in the wild type (horizontal bars) and *mtpt4* mutant (diagonal bars) at P20 only. Values are mean ±standard error, *n*=5. Means followed by the same letter were not significantly different at the *P*<0.05 level (Tukey’s HSD). For (A) data were analysed in more detail at each P level by a Student’s *t*-test comparing the treatment pots (NM of NM,M) and the NM control (NM,NM), which revealed significantly reduced P uptake from pot A in NM,M plants grown at P0 and P20. For (C) different sets of letters (ab, xy) indicate a significant difference (at the *P*<0.05 level, Student’s *t*-test) between M,M and NM,M pots. (This figure is available in colour at *JXB* online.)

Root length-specific P uptake from pot A increased significantly with increasing soil P addition for the non-mycorrhizal roots (i.e., NM,M and NM,NM plants) (Fig. 6A). Furthermore, root length-specific P uptake from pot A was analysed in more detail at each P level by a Student’s *t*-test comparing the treatment pots (NM of NM,M) and the NM control (NM,NM). At P0 and P20, root length-specific P uptake was significantly lower in the NM,M than in the NM,NM plants (*P*<0.05). The wild-type plants had significantly higher root length-specific P uptake at P20 than the *mtpt4* plants in the M,M pots only (Fig. 6B).

The minimum contribution to shoot P by mycorrhizas in pot B decreased significantly with increasing soil P addition in wild-type plants (Fig. 6C). Furthermore, when Student’s *t*-tests were used to compare between NM,M and M,M pots at each P level separately, the minimum contribution to shoot P content was significantly higher in the NM,M pots than in the M,M pots, for the P0 and P20 treatments.

### Gene expression

**Fig. 7.**
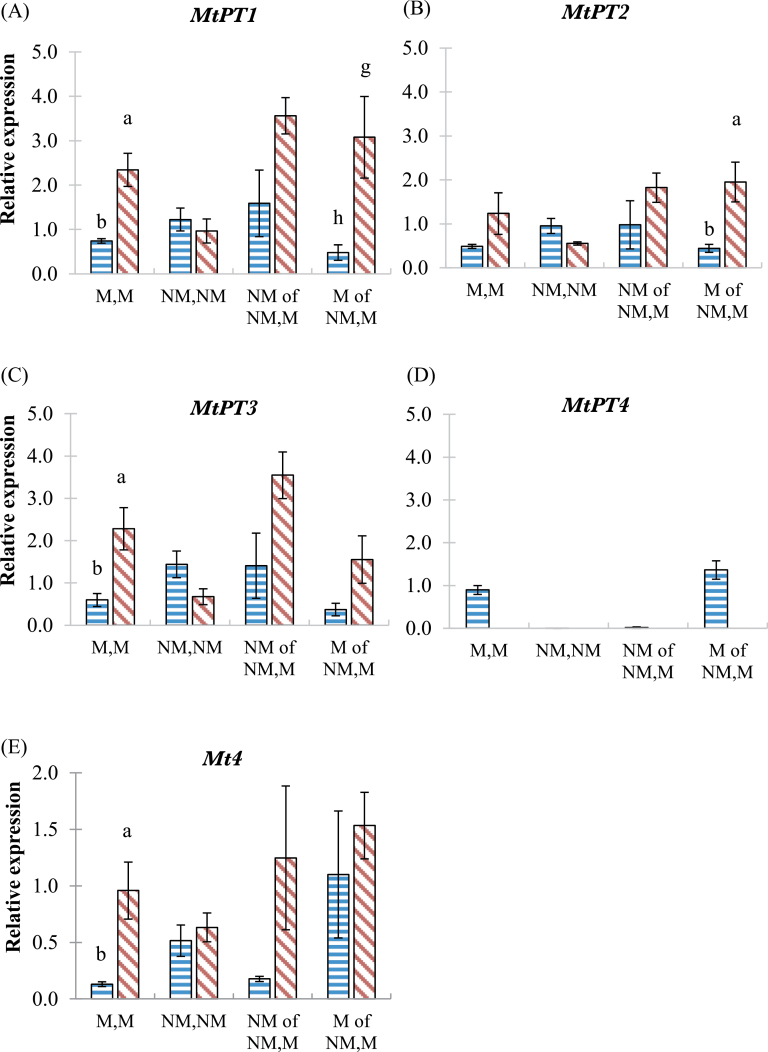
Relative gene expression in roots of wild-type and *mtpt4* plants. Expression of direct Pi transporter genes (A–C) *MtPT1, MtPT2*, and *MtPT3* mycorrhiza-induced Pi transporter gene (D) *MtPT4*, and PSI gene (E) *Mt4* in the wild type (horizontal bars) and *mtpt4* mutant (diagonal bars) at P20 only. Values are the mean ±standard error, *n*=3 or 4. Letters (ab, gh) denote a significant difference (at the *P*<0.05 level, Student’s *t*-test) between genotypes. See main text for further information. (This figure is available in colour at *JXB* online.)

The expression of the direct Pi transporter genes (*MtPT1*, *MtPT2*, and *MtPT3*) in wild-type and *mtpt4* genotypes in the colonized root halves at P20 were evaluated by real-time quantitative PCR, then the results were compared using Student’s *t*-test for each gene. In the M,M pots and in the colonized root half of the NM,M pots, *MtPT1*, *MtPT2*, and *MtPT3* expression was higher in the *mtpt4* roots than in the wild-type roots, in most cases significantly (Fig. 7A–C).

In the non-mycorrhizal control (NM,NM) and treatment (NM of NM,M) pots, there were no significant differences in any of the Pi transporter genes between genotypes (Fig. 7A–C). In the *mtpt4* roots, the expression of *MtPT1*, *MtPT2*, and *MtPT3* in non-mycorrhizal roots of the NM,M treatment was significantly higher than in the non-mycorrhizal control (NM,NM) treatment, whereas this was not the case in wild-type plants. Furthermore, the mycorrhizal Pi transporter gene *MtPT4* was expressed in the mycorrhizal roots of wild-type plants, but not in *mtpt4* mutant roots (Fig. 7D).

Expression of *Mt4* in the mycorrhizal control pots (M,M) was significantly higher in the *mtpt4* genotype than in the wild type. Expression of *Mt4* in other pots (NM,NM and NM,M) was not significantly different between the genotypes, but the expression of *Mt4* in the non-colonized root half in NM,M pots was substantially higher in the *mtpt4* mutant (Fig. 7E).

## Discussion

### The effect of mycorrhizal colonization of one root half on local and distal DPU expression and activity

This work provides molecular but not physiological confirmation of hypothesis (i) that localized root colonization by AM fungi reduces DPU activity locally but not in a distal, non-colonized patch of root. Root length-specific shoot P uptake from a non-colonized root half was actually lower in NM,M pots than in completely non-colonized wild-type plants. However, the magnitude of this systemic AM-induced suppression of DPU activity was much lower than the local suppression in AM roots, where at least 80% of the P taken up was via the MPU at low soil P levels. Considering that up to 100% of a plant’s P can be delivered via the MPU (Smith *et al.*, 2003, 2004), the actual activity of the MPU in the wild-type plants was probably higher than the estimates presented here, as MPU activity may have been ‘hidden’ due to down-regulation of the DPU in AM roots.

The expression of the DPU transporter genes (*MtPT1*, *MtPT2*, and *MtPT3*) did not concur with the physiological data as wild-type plants grown at P20 had similar levels of gene expression in non-colonized plants to those in the non-colonized half of NM,M plants (also seen at P0; data not shown). This suggests that a colonized root half did not exert a long-distance effect upon DPU transporter expression. In contrast, AM colonization had a strong local effect on the DPU and MPU transporter genes. Here, expression of DPU transporters decreased relative to their expression in non-colonized plants, as reported previously (H. Liu *et al.*, 1998), and the MPU transporter gene was expressed as expected (Harrison *et al.*, 2002; Javot *et al.*, 2007). Effects of root colonization on expression levels differed in the *mtpt4* roots, where all DPU transporter genes were expressed similarly in non-mycorrhizal and mycorrhizal roots, but at a higher level than in non-mycorrhizal wild-type roots. The finding that a colonized root half influenced gene expression locally but not at long distance might be related to a local increase in root P concentrations, rather than AM colonization directly. Hence, the effect was absent (and even opposite) in *mtpt4* roots that were colonized but did not transfer mycorrhiza-derived P to the plant. These results with *mtpt4* confirm hypothesis (ii) that mycorrhiza-induced effects on DPU activity can be separated into P-derived and direct AM effects by comparing the wild-type and *mtpt4* genotypes. It was previously suggested by Grønlund *et al.* (2013) that mycorrhizal colonization has a limited long-distance effect upon DPU, which is in agreement with the present findings in terms of gene expression. Thus, although an active MPU in one half of the root system had some impact on DPU activity in the other half of the system, this was not related to gene expression. There is growing evidence that P transporter gene expression is not always well correlated with P uptake activity (or flux) (C. Liu *et al.*, 1998; Rae *et al.*, 2004; Grace *et al.*, 2009; Smith *et al.*, 2009; Grønlund *et al.*, 2013; Facelli *et al.*, 2014); this could be due to post-transcriptional and/or post-translational regulation of the P uptake pathways.

To further explore the effect of AM colonization on plant P uptake and nutrition, expression of *Mt4*, a PSI gene in *M. truncatula*, and PO_4_ concentration in shoots was investigated. Expression of *Mt4* was inversely proportional to the PO_4_ concentration of shoots, as expected (Burleigh and Harrison, 1997, 1998; Branscheid *et al.*, 2010). Furthermore, expression of *Mt4* was similar between the wild-type and *mtpt4* mutant genotypes in non-mycorrhizal control pots. In colonized root halves, the *Mt4* expression was similar to that in non-mycorrhizal roots in *mtpt4* mutant plants, while it was suppressed in the wild-type plants, probably reflecting the local increase in PO_4_ root concentrations (Burleigh and Harrison, 1998), as shoot PO_4_ concentrations were similar for wild-type and *mtpt4* NM,M plants.

In conclusion, there were both physiological and molecular effects of an inactive mycorrhizal P uptake pathway (*mtpt4*) upon the DPU, which seems to correspond to local changes of P/Pi concentrations in colonized roots in a functional symbiosis. Interestingly, the expression of the DPU transporter genes in *mtpt4* roots was higher in the AM plants, both NM,M and M,M, than in NM,NM plants and in wild-type plants of all treatments. This indicates that the mutant plants compensate for the non-functional MPU, and subsequent loss of contribution of P by the MPU.

### The effect of mycorrhizal colonization of one root half on root distribution and P nutrition

This work confirms hypothesis (iii) that colonized and non-colonized patches of root will experience differences in root growth and P nutrition. Specifically, root biomass was preferentially allocated to the colonized root half rather than the non-colonized root half in the wild-type plants where the P concentrations were also higher, but this was not the case in the *mtpt4* plants. Previous split-root experiments also found greater root biomass and P concentration in the mycorrhizal side of the pot compared with the non-mycorrhizal side (Grønlund *et al.*, 2013), as well as greater amounts of photosynthate transported to the mycorrhizal half of the split pot from the shoots (Koch and Johnson, 1984). In general terms, mycorrhizal colonization increases root branching and root length (Hodge *et al.*, 2009). The lower root length in mycorrhizal than in non-mycorrhizal plants in this work (M,M versus NM,NM) and in several previous studies (e.g. Ravnskov and Jakobsen, 1995; Olsson *et al.*, 1997) was probably caused by fungal competition for plant carbon in the absence of a mycorrhizal growth response, but a high root colonization, at 20mg and 50mg P kg^–1^.

In contrast to the wild-type, root biomass and root length in the *mtpt4* mutant plants was higher in the non-colonized than in the colonized root halves. The higher expression of *Mt4* and DPU *MtPT* genes in colonized roots compared with non-mycorrhizal roots suggests an increased sensing of Pi starvation in the colonized *mtpt4* root half, although P concentrations were the same for all non-mycorrhizal and mycorrhizal root halves, which fits with only the DPU being active.

Although the colonized *mtpt4* roots did not take up Pi via the MPU, the plant still supported colonization by AM fungi, which would have come at a carbon cost to the plant (Smith *et al.*, 2010), and may explain the observed reduction in shoot and root growth in colonized *mtpt4* plants compared with the wild type. Furthermore, the *mtpt4* plants may have reduced investment in the colonized root half in favour of the non-colonized root half, which would be providing a return on the investment (in terms of Pi uptake).

Proper functioning of *MtPT4* is critical to mycorrhiza-mediated Pi uptake, and arbuscules of colonized *mtpt4* roots experience premature degeneration and death when MtPT4 is inactivated (Javot *et al.*, 2007). However, premature arbuscule death can be suppressed by N limitation (Javot *et al.*, 2011). The *mtpt4* plants in this study had shoot N concentrations >3% dry weight in all treatments (data not shown), which suggested that they were not N limited (Marschner and Marschner, 2012). The arbuscules may therefore have been experiencing premature degeneration in the *mtpt4* roots, and this is supported by the lower percentage of arbuscules in the mutant compared with wild-type roots. However, the colonized *mtpt4* roots, although defective in MPU Pi uptake, were still producing external hyphae to a greater extent than previously reported (Javot *et al.*, 2007), although at lower densities than the wild-type. This phenotype observed in the present study will require further work to be confirmed in light of the differences with the work of Javot *et al.* (2007).

In conclusion, partial colonization of roots by AM fungi had marked effects on the root growth distribution and on Pi uptake locally in the colonized root system. This highlights the plasticity of roots in response to AM colonization, where most research on root plasticity has focused on it in response to soil nutrient patches. Root plasticity in response to AM colonization could confer greater competitive ability for the plant, in terms of increased Pi uptake, and more effective allocation of root biomass and length.

There was physiological evidence that colonization by AM fungi had a limited, long-distance impact upon DPU activity, but this was not supported by gene expression data. This highlights the growing disconnect between gene expression data and actual activity, and, thus, the need to include both physiological and molecular methods, and for further studies on up- and downstream regulatory events.

Comparison of the results from wild-type and *mtpt4* mutant plants indicated that a major factor in the effect of AM colonization on the DPU could rely on the local changes in root P concentrations in an active symbiosis, as *mtpt4* mycorrhizal roots behaved mostly like non-mycorrhizal roots, although they were colonized. Furthermore, the *mtpt4* plants seemed to have additional sensing of the carbon cost of the non-functional AM symbiosis, as photosynthates were allocated to the non-colonized root half.

## Supplementary data

Supplementary data are available at *JXB* online.

Figure S1. Inorganic phosphate (PO_4_) concentration in shoots.

Table S1. ANOVA summary table for all response variables.

Table S2. Mycorrhizal colonization (% of root length) of *mtpt4* mutant genotype, grown at 20mg P kg^-1^.

Supplementary Data

## References

[CIT0001] Balzergue C Puech-Pagès V Bécard G Rochange SF 2011 The regulation of arbuscular mycorrhizal symbiosis by phosphate in pea involves early and systemic signalling events. Journal of Experimental Botany 62, 1049–1060. 21045005 10.1093/jxb/erq335 PMC3022399

[CIT0002] Branscheid A Sieh D Pant BD May P Devers EA Elkrog A Schauser L Scheible W-R Krajinski F 2010 Expression pattern suggests a role of MiR399 in the regulation of the cellular response to local Pi increase during arbuscular mycorrhizal symbiosis. Molecular Plant-Microbe Interactions 23, 915–926. 20521954 10.1094/MPMI-23-7-0915

[CIT0003] Breuillin F Schramm J Hajirezaei M 2010 Phosphate systemically inhibits development of arbuscular mycorrhiza in Petunia hybrida and represses genes involved in mycorrhizal functioning. The Plant Journal 64, 1002–1017. 21143680 10.1111/j.1365-313X.2010.04385.x

[CIT0004] Bucher M 2007 Functional biology of plant phosphate uptake at root and mycorrhiza interfaces. New Phytologist 173, 11–26. 17176390 10.1111/j.1469-8137.2006.01935.x

[CIT0005] Bucher M Rausch C Daram P 2001 Molecular and biochemical mechanisms of phosphorus uptake into plants. Journal of Plant Nutrition and Soil Science 164, 209–217.

[CIT0006] Burleigh SH Harrison MJ 1997 A novel gene whose expression in Medicago truncatula roots is suppressed in response to colonization by vesicular-arbuscular mycorrhizal (VAM) fungi and to phosphate nutrition. Plant Molecular Biology 34, 199–208. 9207836 10.1023/a:1005841119665

[CIT0007] Burleigh SH Harrison MJ 1999 The down-regulation of Mt4-like genes by phosphate fertilization occurs systemically and involves phosphate translocation to the shoots. Plant Physiology 119, 241–248. 9880366 10.1104/pp.119.1.241 PMC32226

[CIT0008] Burleigh SJ Harrison MJ 1998 Characterization of the Mt4 gene from Medicago truncatula. Gene 216, 47–53. 9714729 10.1016/s0378-1119(98)00326-6

[CIT0009] Chiou T-J Liu H Harrison MJ 2001 The spatial expression patterns of a phosphate transporter (MtPT1) from Medicago truncatula indicate a role in phosphate transport at the root/soil interface. The Plant Journal 25, 281–293. 11208020 10.1046/j.1365-313x.2001.00963.x

[CIT0010] Christophersen HM Smith FA Smith SE 2009 Arbuscular mycorrhizal colonization reduces arsenate uptake in barley via downregulation of transporters in the direct epidermal phosphate uptake pathway. New Phytologist 184, 962–974. 19754635 10.1111/j.1469-8137.2009.03009.x

[CIT0011] Cordell D Drangert JO White S 2009 The story of phosphorus: global food security and food for thought. Global Environmental Change-Human and Policy Dimensions 19, 292–305.

[CIT0012] Epstein E Bloom AJ 2005 Mineral nutrition of plants: principles and perspectives. Sunderland, MA: Sinauer Associates.

[CIT0013] Facelli E Duan T Smith SE Christophersen HM Facelli JM Smith FA 2014 Opening the black box: outcomes of interactions between arbuscular mycorrhizal (AM) and non-host genotypes of Medicago depend on fungal identity, interplay between P uptake pathways and external P supply. Plant, Cell and Environment 37, 1382–1392. 10.1111/pce.12237 24236504

[CIT0014] Franco-Zorrilla JM González E Bustos R Linhares F Leyva A Paz-Ares J 2004 The transcriptional control of plant responses to phosphate limitation. Journal of Experimental Botany 55, 285–293. 14718495 10.1093/jxb/erh009

[CIT0015] Gilbert N 2009 Environment: the disappearing nutrient. Nature 461, 716–718. 19812648 10.1038/461716a

[CIT0016] Glassop D Smith S Smith F 2005 Cereal phosphate transporters associated with the mycorrhizal pathway of phosphate uptake into roots. Planta 222, 688–698. 16133217 10.1007/s00425-005-0015-0

[CIT0017] Grace EJ Cotsaftis O Tester M Smith FA Smith SE 2009 Arbuscular mycorrhizal inhibition of growth in barley cannot be attributed to extent of colonization, fungal phosphorus uptake or effects on expression of plant phosphate transporter genes. New Phytologist 181, 938–949. 19140934 10.1111/j.1469-8137.2008.02720.x

[CIT0018] Grønlund M Albrechtsen M Johansen IE Hammer EC Nielsen TH Jakobsen I 2013 The interplay between P uptake pathways in mycorrhizal peas: a combined physiological and gene-silencing approach. Physiologia Plantarum 149, 234–248. 23387980 10.1111/ppl.12030

[CIT0019] Grunwald U Guo W Fischer K Isayenkov S Ludwig-Müller J Hause B Yan X Küster H Franken P 2009 Overlapping expression patterns and differential transcript levels of phosphate transporter genes in arbuscular mycorrhizal, Pi-fertilised and phytohormone-treated Medicago truncatula roots. Planta 229, 1023–1034. 19169704 10.1007/s00425-008-0877-z PMC2757622

[CIT0020] Harrison MJ 2005 Signaling in the arbuscular mycorrhizal symbiosis. Annual Review of Microbiology 59, 19–42. 10.1146/annurev.micro.58.030603.123749 16153162

[CIT0021] Harrison MJ Dewbre GR Liu J 2002 A phosphate transporter from Medicago truncatula involved in the acquisition of phosphate released by arbuscular mycorrhizal fungi. The Plant Cell 14, 2413–2429. 12368495 10.1105/tpc.004861 PMC151226

[CIT0022] Hodge A Berta G Doussan C Merchan F Crespi M 2009 Plant root growth, architecture and function. Plant and Soil 321, 153–187.

[CIT0023] Jakobsen I Abbott LK Robson AD 1992 External hyphae of vesicular-arbuscular mycorrhizal fungi associated with Trifolium subterraneum L 1. Spread of hyphae and phosphorus inflow into roots. New Phytologist 120, 371–380.

[CIT0024] Javot H Penmetsa RV Breuillin F Bhattarai KK Noar RD Gomez SK Zhang Q Cook DR Harrison MJ 2011 Medicago truncatula mtpt4 mutants reveal a role for nitrogen in the regulation of arbuscule degeneration in arbuscular mycorrhizal symbiosis. The Plant Journal 68, 954–965. 21848683 10.1111/j.1365-313X.2011.04746.x

[CIT0025] Javot H Penmetsa RV Terzaghi N Cook DR Harrison MJ 2007 A Medicago truncatula phosphate transporter indispensable for the arbuscular mycorrhizal symbiosis. Proceedings of the National Academy of Sciences, USA 104, 1720–1725. 10.1073/pnas.0608136104 PMC1785290 17242358

[CIT0026] Koch KE Johnson CR 1984 Photosynthate partitioning in split-root citrus seedlings with mycorrhizal and nonmycorrhizal root systems. Plant Physiology 75, 26–30. 16663589 10.1104/pp.75.1.26 PMC1066828

[CIT0027] Kormanik P McGraw A 1982 Quantification of vesicular-arbuscular mycorrhizae in plant roots. In: Schenck N, ed. Methods and principles of mycorrhizal research. St Paul, MN: American Phytopathological Society, 37–45.

[CIT0028] Lambers H Brundrett MC Raven JA Hopper SD 2010 Plant mineral nutrition in ancient landscapes: high plant species diversity on infertile soils is linked to functional diversity for nutritional strategies. Plant and Soil 334, 11–31.

[CIT0029] Lanzetta PA Alvarez LJ Reinach PS Candia OA 1979 An improved assay for nanomole amounts of inorganic phosphate. Analytical Biochemistry 100, 95–97. 161695 10.1016/0003-2697(79)90115-5

[CIT0030] Liu C Muchhal US Uthappa M Kononowicz AK Raghothama KG 1998 Tomato phosphate transporter genes are differentially regulated in plant tissues by phosphorus. Plant Physiology 116, 91–99. 9449838 10.1104/pp.116.1.91 PMC35191

[CIT0031] Liu H Trieu AT Blaylock LA Harrison MJ 1998 Cloning and characterization of two phosphate transporters from Medicago truncatula roots: regulation in response to phosphate and to colonization by arbuscular mycorrhizal (AM) fungi. Molecular Plant-Microbe Interactions 11, 14–22. 9425684 10.1094/MPMI.1998.11.1.14

[CIT0032] Liu J Versaw WK Pumplin N Gomez SK Blaylock LA Harrison MJ 2008 Closely related members of the Medicago truncatula PHT1 phosphate transporter gene family encode phosphate transporters with distinct biochemical activities. Journal of Biological Chemistry 283, 24673–24681. 18596039 10.1074/jbc.M802695200 PMC3259825

[CIT0033] López-Arredondo DL Leyva-González MA González-Morales SI López-Bucio J Herrera-Estrella L 2014 Phosphate nutrition: improving low-phosphate tolerance in crops. Annual Review of Plant Biology 65, 95–123. 10.1146/annurev-arplant-050213-035949 24579991

[CIT0034] Marschner H Dell B 1994 Nutrient uptake in mycorrhizal symbiosis. Plant and Soil 159, 89–102.

[CIT0035] Marschner H Marschner P 2012 Marschner’s mineral nutrition of higher plants. New York: Academic Press.

[CIT0036] Martín AC Del Pozo JC Iglesias J Rubio V Solano R De La Peña A Leyva A Paz-Ares J 2000 Influence of cytokinins on the expression of phosphate starvation responsive genes in Arabidopsis. The Plant Journal 24, 559–567. 11123795 10.1046/j.1365-313x.2000.00893.x

[CIT0037] McGonigle T Miller M Evans D Fairchild G Swan J 1990 A new method which gives an objective measure of colonization of roots by vesicular-arbuscular mycorrhizal fungi. New Phytologist 115, 495–501. 10.1111/j.1469-8137.1990.tb00476.x 33874272

[CIT0038] Merrild MP Ambus P Rosendahl S Jakobsen I 2013 Common arbuscular mycorrhizal networks amplify competition for phosphorus between seedlings and established plants. New Phytologist 200, 229–240. 23738787 10.1111/nph.12351

[CIT0039] Nagy R Drissner D Amrhein N Jakobsen I Bucher M 2009 Mycorrhizal phosphate uptake pathway in tomato is phosphorus-repressible and transcriptionally regulated. New Phytologist 181, 950–959. 19140941 10.1111/j.1469-8137.2008.02721.x

[CIT0040] Newman EI 1966 A method of estimating total length of root in a sample. Journal of Applied Ecology 3, 139–145.

[CIT0041] Olsen S Cole C Watanabe F Dean L 1954 Estimation of available phosphorus in soils by extraction with sodium bicarbonate. Washington, DC: USDA Circular 939.

[CIT0042] Olsson PA Baath E Jakobsen I 1997 Phosphorus effects on the mycelium and storage structures of an arbuscular mycorrhizal fungus as studied in the soil and roots by analysis of fatty acid signatures. Applied and Environmental Microbiology 63, 3531–3538. 16535691 10.1128/aem.63.9.3531-3538.1997 PMC1389247

[CIT0043] Paszkowski U Kroken S Roux C Briggs SP 2002 Rice phosphate transporters include an evolutionarily divergent gene specifically activated in arbuscular mycorrhizal symbiosis. Proceedings of the National Academy of Sciences, USA 99, 13324–13329. 10.1073/pnas.202474599 PMC130632 12271140

[CIT0044] Péret B Clément M Nussaume L Desnos T 2011 Root developmental adaptation to phosphate starvation: better safe than sorry. Trends in Plant Science 16, 442–450. 21684794 10.1016/j.tplants.2011.05.006

[CIT0045] Poulsen KH Nagy R Gao LL Smith SE Bucher M Smith FA Jakobsen I 2005 Physiological and molecular evidence for Pi uptake via the symbiotic pathway in a reduced mycorrhizal colonization mutant in tomato associated with a compatible fungus. New Phytologist 168, 445–453. 16219083 10.1111/j.1469-8137.2005.01523.x

[CIT0046] Rae AL Jarmey JM Mudge SR Smith FW 2004 Over-expression of a high-affinity phosphate transporter in transgenic barley plants does not enhance phosphate uptake rates. Functional Plant Biology 31, 141–148. 10.1071/FP03159 32688886

[CIT0047] Raghothama KG 1999 Phosphate acquisition. Annual Review of Plant Physiology and Plant Molecular Biology 50, 665–693. 10.1146/annurev.arplant.50.1.665 15012223

[CIT0048] Ravnskov S Jakobsen I 1995 Functional compatibility in arbuscular mycorrhizas measured as hyphal P transport to the plant. New Phytologist 129, 611–618.

[CIT0049] Rouached H Arpat AB Poirier Y 2010 Regulation of phosphate starvation responses in plants: signaling players and cross-talks. Molecular Plant 3, 288–299. 20142416 10.1093/mp/ssp120

[CIT0050] Schachtman DP Reid RJ Ayling SM 1998 Phosphorus uptake by plants: from soil to cell. Plant Physiology 116, 447–453. 9490752 10.1104/pp.116.2.447 PMC1539172

[CIT0051] Schüßler A Walker C 2010 The *Glomeromycota*. A species list with new families and new genera. The Royal Botanic Garden Edinburgh.

[CIT0052] Smith FA Grace EJ Smith SE 2009 More than a carbon economy: nutrient trade and ecological sustainability in facultative arbuscular mycorrhizal symbioses. New Phytologist 182, 347–358. 19207688 10.1111/j.1469-8137.2008.02753.x

[CIT0053] Smith SE Facelli E Pope S Smith FA 2010 Plant performance in stressful environments: interpreting new and established knowledge of the roles of arbuscular mycorrhizas. Plant and Soil 326, 3–20.

[CIT0054] Smith SE Jakobsen I Grønlund M Smith FA 2011 Roles of arbuscular mycorrhizas in plant phosphorus nutrition: interactions between pathways of phosphorus uptake in arbuscular mycorrhizal roots have important implications for understanding and manipulating plant phosphorus acquisition. Plant Physiology 156, 1050–1057. 21467213 10.1104/pp.111.174581 PMC3135927

[CIT0055] Smith SE Read DJ 2008 Mycorrhizal symbiosis. New York: Academic Press.

[CIT0056] Smith SE Smith FA Jakobsen I 2003 Mycorrhizal fungi can dominate phosphate supply to plants irrespective of growth responses. Plant Physiology 133, 16–20. 12970469 10.1104/pp.103.024380 PMC1540331

[CIT0057] Smith SE Smith FA Jakobsen I 2004 Functional diversity in arbuscular mycorrhizal (AM) symbioses: the contribution of the mycorrhizal P uptake pathway is not correlated with mycorrhizal responses in growth or total P uptake. New Phytologist 162, 511–524.

[CIT0058] Xiao K Liu J Dewbre G Harrison M Wang Z 2006 Isolation and characterization of root-specific phosphate transporter promoters from Medicago truncatula. Plant Biology 8, 439–449. 16917979 10.1055/s-2005-873053

