# Supplementary material for: Local and distal effects of arbuscular mycorrhizal colonization on direct pathway Pi uptake and root growth in Medicago truncatula
Source: J Exp Bot. 2015 May 4;66(13):4061–73. doi: 10.1093/jxb/erv202 (PMC4473995; doi:10.1093/jxb/erv202)
Supplement: Supplementary Data [file supp_66_13_4061__index.html]

Local and distal effects of arbuscular mycorrhizal colonization on direct pathway Pi uptake and root growth in Medicago truncatula — Local and distal effects of arbuscular mycorrhizal colonization on direct pathway Pi uptake and root growth in Medicago truncatula — Supplementary Data 

# Local and distal effects of arbuscular mycorrhizal colonization on direct pathway Pi uptake and root growth in *Medicago truncatula*

## Supplementary Data

Data files

**Files in this Data Supplement:**

- Supplementary Data - Supplementary Data
