# Supplementary material for: Local and distal effects of arbuscular mycorrhizal colonization on direct pathway Pi uptake and root growth in Medicago truncatula
Source: J Exp Bot. 2015 May 4;66(13):4061–73. doi: 10.1093/jxb/erv202 (PMC4473995; doi:10.1093/jxb/erv202)
Supplement: Supplementary Data [file supp_erv202_jexbot144204_file001.pdf]

Supplementary material

Table S1. ANOVA summary table for all response variables.

(A)

	P	*C*	*P*C*
Colonisation (pot A)	ns	***	ns
Colonisation (pot B)	**	***	*
SDW	***	ns	**
RDW (total)	***	ns	*
Root length (total)	***	**	*
Shoot P content	***	ns	***
Root P content (total)	***	***	***
Root P conc. (pot A)	***	***	***
Root P conc. (pot B)	***	***	***
Pot A P uptake	**	**	***
Shoot PO_4 conc.	***	**	ns

(B)

	G	*C*	*G*C*
Colonisation (pot A)	ns	***	ns
Colonisation (pot B)	***	***	***
SDW	***	ns	*
RDW (total)	**	ns	ns
Root length (total)	ns	**	ns
Shoot P content	***	ns	***
Root P content (total)	***	**	***
Root P conc. (pot A)	***	***	***
Root P conc. (pot B)	***	***	***
Pot A P uptake	**	***	***
Shoot PO_4 conc.	*	ns	*

Factors in the analysis were (A) *P* and *C*, (B) *G* and *C*. Both main effects and interaction terms are indicated. *P* Soil P Addition, *C* Pot Configuration, *G* Genotype. *$P<0.05$; **$P<0.01$; ***$P<0.0001$.

Table S2. Mycorrhizal colonisation (% of root length) of *mtpt4* mutant genotype, grown at 20 mg P kg^{-1}. Values are mean ± standard error, n=5. Means followed by the same letter were not significantly different at the P<0.05 level (Tukey's HSD).

		M,M	NM,M	NM,NM
Pot A	mean	35.6	0	0
	s.e.	4.9	0	0
Pot B	mean	35.2	31.7	0
	s.e.	5.9	3.7	0

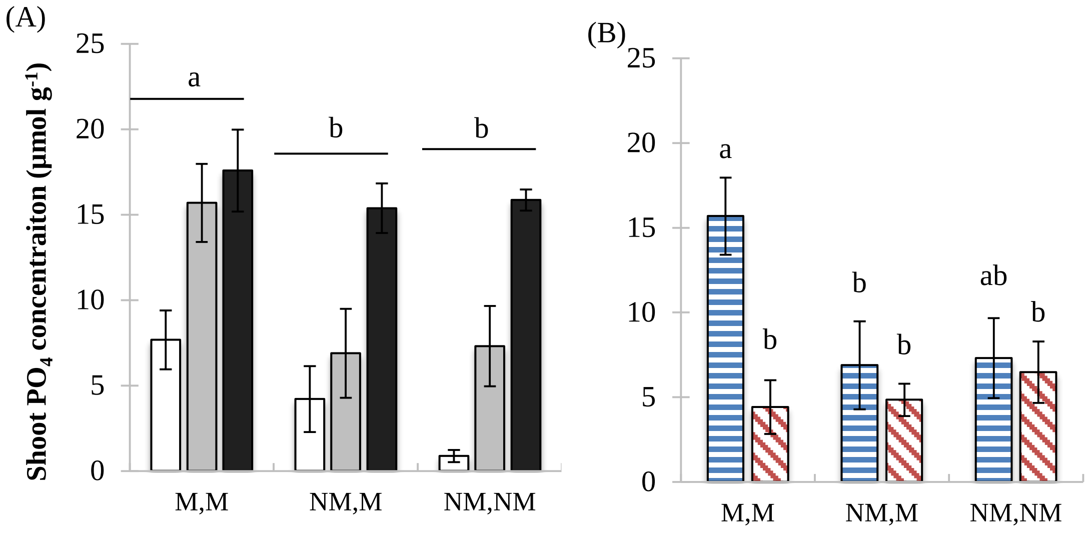

Figure S1. Inorganic phosphate (PO_4) concentration in shoots.

PO_4 concentration (µmol g^{-1}) in the fresh shoots of (A) wild-type plants at three soil P additions: 0 (white bars), 20 (grey bars) and 50 (black bars) mg P kg^{-1}, and in (B) wild-type (horizontal bars) and *mtpt4* mutant (diagonal bars) at P20 only. Values are mean ± standard error, n=4. Means followed by the same letter were not significantly different at the P<0.05 level (Tukey's HSD). See text for details of statistical analysis.
